# Chronic contained rupture of abdominal aortic aneurism complicated with aortic occlusion: a case report

**DOI:** 10.1186/s40792-019-0654-1

**Published:** 2019-06-20

**Authors:** Azuma Tabayashi, Takeshi Kamada, Akihiko Abiko, Ryoichi Tanaka, Hajime Kin

**Affiliations:** 1 Department of Cardiovascular Surgery, Iwate Medical University, Memorial Heart Center, 19-1 Uchimaru, Morioka, Iwate 020-8505 Japan; 2 Department of Cardiology, Iwate Medical University, 19-1 Uchimaru, Morioka, Iwate 020-8505 Japan; 3 Department of Oral and Maxillofacial Surgery/Department of Radiology, Iwate Medical University, 19-1 Uchimaru, Morioka, Iwate 020-8505 Japan

**Keywords:** Chronic contained rupture, Retroperitoneal tumor, Abdominal aortic occlusion

## Abstract

**Background:**

Chronic contained rupture is a subtype of an abdominal aortic aneurysm rupture. Its diagnosis is sometimes difficult due to lack of typical symptoms. We would like to report the challenge of diagnosing chronic contained rupture of abdominal aortic aneurysm with a retroperitoneal tumor.

**Case presentation:**

A 60-year-old man reported perceived lower abdominal pain 7 months earlier that spontaneously remitted. A contrast-enhanced computed tomography (CT) indicated an abdominal aortic aneurysm and a mass lesion surrounding the abdominal aorta and iliac arteries. Fluorine-18-fluorodeoxyglucose positron emission tomography (FDG-PET) showed an increased accumulation of FDG in the margin of the lesion, indicating a retroperitoneal tumor. A CT-guided biopsy revealed only retroperitoneal fibrous tissue with chronic inflammation. We were thus unable to reach a definitive diagnosis. At 1 month after the initial examination, intermittent claudication was newly observed. A follow-up contrast-enhanced CT scan revealed abdominal aortic occlusion. Mass resection and bypass surgery were performed for diagnosis and treatment. Intraoperative and pathological findings led to the diagnosis of chronic contained rupture of an abdominal aortic aneurysm. The patient was discharged 19 days after surgery.

**Conclusion:**

The mass peripheral to the abdominal aorta should be considered the possibility not only of tumor but also of chronic contained rupture of an abdominal aortic aneurysm.

## Background

Chronic contained rupture of an abdominal aortic aneurysm (CCR-AAA) is a well-known subtype of a AAA rupture. Its chronic course precludes typical clinical findings as seen in a AAA rupture, such as severe abdominal pain or hemodynamic changes. Moreover, although contrast-enhanced computed tomography (CT), fluorine-18-fluorodeoxyglucose positron emission tomography (FDG-PET), and magnetic resonance imaging (MRI) aid diagnosis, they fail to distinguish CCR-AAA from a retroperitoneal tumor [1, 2]. Herein, we report the challenge of diagnosing CCR-AAA with a retroperitoneal tumor.

## Case presentation

**Fig. 1:**
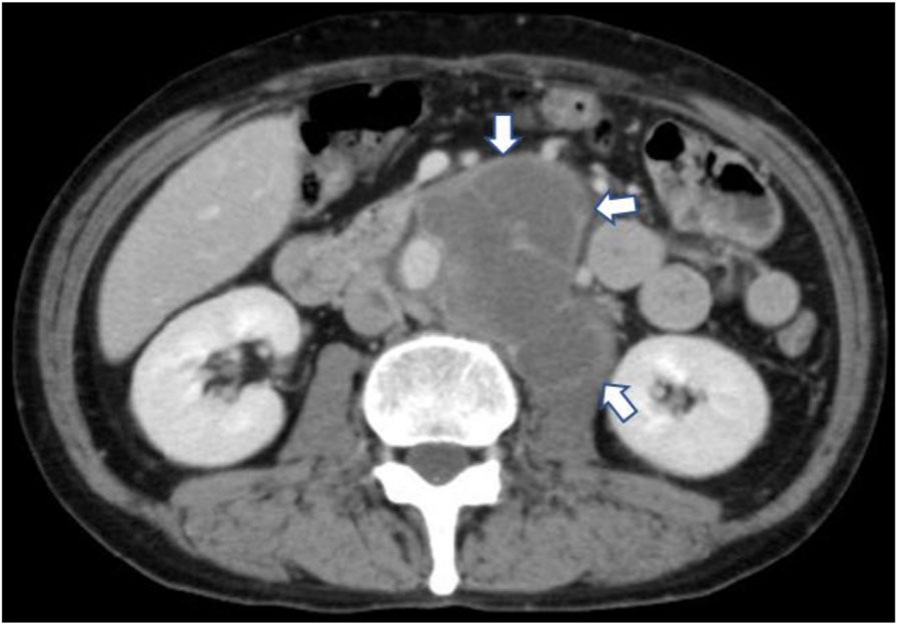
Enhanced computed tomography at initial examination showing the large mass lesion along with the abdominal aorta. The margin of the mass lesion is enhanced (arrow)

**Fig. 2:**
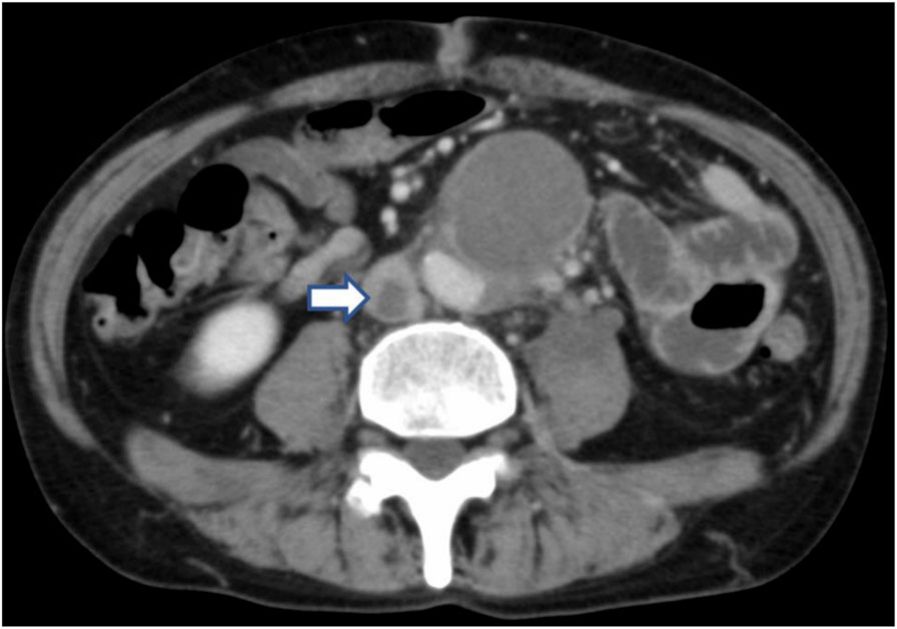
Enhanced computed tomography at initial examination showing the 10 mm thrombus in the inferior vena cava (arrow)

The patient was a 60-year-old man. Abdominal ultrasonography indicated that he had an abdominal mass. He reported having perceived lower abdominal pain 7 months before the initial examination, but it spontaneously remitted. The patient had no fever nor any history of conditions such as hypertension. A blood test revealed that he did not have anemia, thrombocytopenia, or coagulation abnormalities. The patient demonstrated slightly elevated levels of carcinoembryonic antigen (4.7 ng/ml) and soluble interleukin-2 receptor (603 U/ml), tumor markers that are elevated in gastrointestinal cancers and malignant lymphoma. Contrast-enhanced CT showed a 33× 31 mm abdominal aortic aneurysm and a 106 × 81 mm mass peripheral to the abdominal aorta. The mass did not feature a contrast effect, and its margin was enhanced in the venous phase (Fig. 1). Sigmoid wall thickening and inferior vena cava thrombosis were also observed (Fig. 2). We suspected the patient had a neoplastic disease, such as a malignant lymphoma or sigmoid colon cancer. Detailed examinations were consequently performed, and direct oral anticoagulant therapy was initiated to treat the inferior vena cava thrombosis.

The FDG-PET revealed an abnormal accumulation of FDG in the margin of the mass and the sigmoid colon, but not in any other organs. Colonoscopy showed only mild inflammation in the sigmoid colon, and a sigmoid colon biopsy revealed no significant findings. Chronic inflamed fibrous connective tissue was harvested from the periaortic mass using CT-guided biopsy. There were no findings indicative of infection or neoplastic disease, thus precluding a pathological diagnosis.

**Fig. 3:**
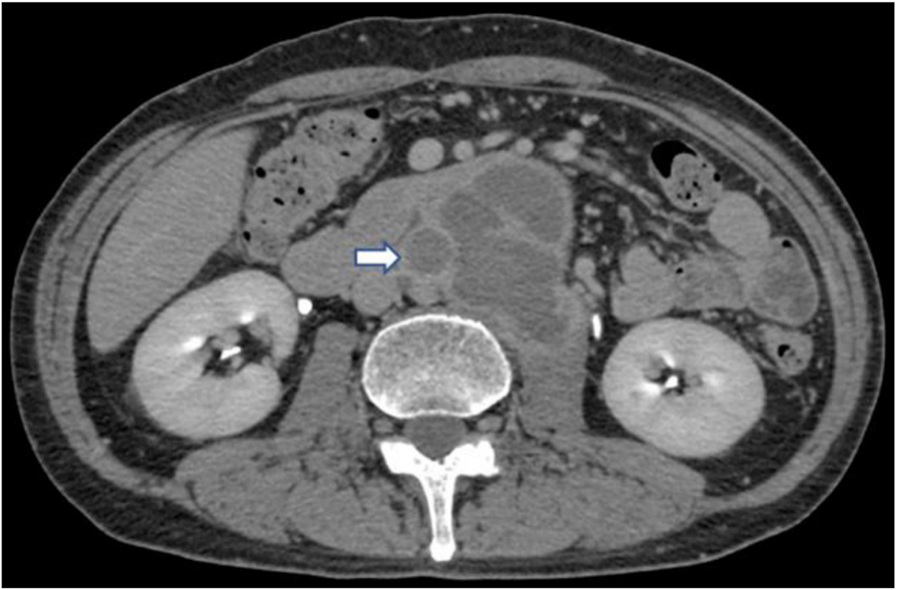
Follow-up enhanced computed tomography at 1 month after the initial examination showing total occlusion of the abdominal aorta (arrow)

At 1 month after the initial examination, intermittent claudication occurred, prompting the performance of follow-up contrast-enhanced CT scanning. On CT scanning, we found an arterial occlusion from the infrarenal abdominal aorta to the bilateral common iliac arteries, the right external iliac artery, and the right internal iliac artery (Fig. 3). These findings indicated the potential progress of the tumor to the aorta. Via a midline incision, biopsies of the mass and the lymph nodes peripheral to the sigmoid colon were obtained. However, these biopsies showed only connective tissue with inflammation and fibrosis and did not yield any significant findings. Since the developments, we elected to resect the mass and perform bypass surgery for definitive diagnosis and treatment after 3 weeks from biopsy.

**Fig. 4:**
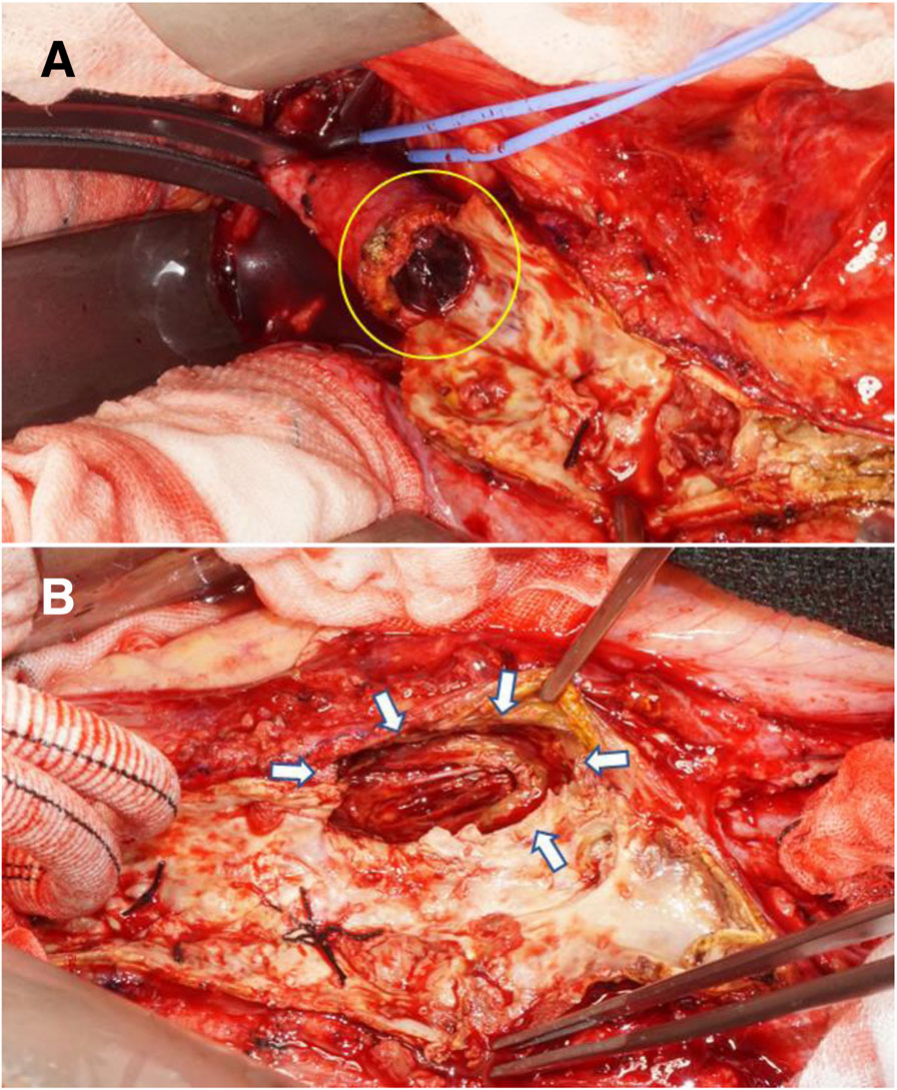
Operative findings. **a** Intraluminal thrombus of the abdominal aorta (ring). **b** Punched-out large defect on the posterior wall (arrow)

Surgery was performed using a transperitoneal approach via a midline incision. Adhesion was observed peripheral to the abdominal aorta, the bilateral iliac arteries, and the retroperitoneal mass. The abdominal aortic lumen was completely occluded by an old thrombus and plaque. Removal of the thrombus revealed a 35-mm intimal defect consistent with the direction of the retroperitoneal mass (Fig. 4). Communication was observed between the intimal defect site and the retroperitoneal mass, which was filled with a partially organized old thrombus. During the intraoperative rapid pathological examination, the tissue inside the mass was diagnosed as a fibrin clot. The tumor tissue was not assessed. The area from the infrarenal abdominal aorta to the right common femoral artery and the left common iliac artery was bypassed using a Y-shaped woven Dacron graft. Pathologic examination showed that the mass tissue was a partially organized fibrin clot, thus agreeing with our intraoperative rapid diagnosis. On the basis of the intraoperative and pathologic findings, the patient was diagnosed with CCR-AAA with abdominal aortic occlusion. His intermittent claudication improved, and he was discharged on day 19 after surgery. Six months after the operation, the retroperitoneal mass disappeared on CT.

## Discussion

A CCR-AAA is a well-known, extremely rare subtype of AAA that was first reported by Szilagyi et al. in 1961 [1–3]. It is characterized by low blood loss and stable hemodynamics, which are due to hemostasis by tissue peripheral to the retroperitoneum when the aortic aneurysm ruptures [4, 5].

The diagnostic criteria for CCR-AAA consist of the following: (1) known AAA; (2) previous pain symptoms; (3) stable condition and normal hematocrit; (4) a CT scan showing a retroperitoneal hematoma; and (5) pathological confirmation of an organized hematoma [1, 2]. Although back pain is common, cases of CCR-AAA present with a wide variety of clinical findings, including cases which are asymptomatic [4–6]. The diagnosis of CCR-AAA thus poses a dilemma. Although the present case met the first three aforementioned criteria, we could not obtain conclusive evidence indicating that the neoplastic lesion observed via the CT was a hematoma, nor could we diagnose CCR-AAA to the exclusion of other diseases. Diseases from which CCR-AAA must be distinguished include retroperitoneal tumor and abscess [1, 2]. CT is a useful modality for diagnosing CCR-AAA; it shows a well-defined soft-tissue density mass, inside which there is often no contrast effect, adjacent to the aortic wall [4]. In addition to CT, FDG-PET is also reported to be effective for the definitive diagnosis of CCR-AAA. Although neoplastic and inflamed lesions typically exhibit accumulation of FDG in the lesion, CCR-AAA is characterized by the accumulation of FDG in the margin of the neoplastic lesion alone and by the absence of accumulation in the abdominal aorta. FDG-PET confirmed such criteria in the present case. However, these findings may also be observed in abscesses and in tumors with central necrosis [2]. Moreover, increased accumulation of FDG was also observed in the sigmoid colon, and tumor markers were elevated. We could not therefore rule out the possibility of neoplastic disease. During the course of the disease, we also suspected invasion by the tumor because of the abdominal aortic occlusion. Although tumor invasion into the aorta is relatively rare, invasion into blood vessels by malignancies, such as sarcomas, should be considered [7, 8].

The fact that CCR-AA is a rare condition further confounds diagnosis. Although there have been several reported cases in recent years of CCR-AAA with vertebral invasion, we could find only one reported case of CCR-AAA with abdominal aortic occlusion [4]. In the report by Yokomuro et al., complication of CCR-AAA by infrarenal abdominal aortic occlusion was confirmed. However, abdominal aortic occlusion had already been observed in the initial examination. Nothing was stated concerning a relationship between the aortic occlusion and CCR-AAA [4].

In the present case, the abdominal aorta was confirmed to be patent at the initial examination, and there were no stenotic lesions. However, a detailed examination at 1 month showed that occlusion had occurred in the course of CCR-AAA. Although antiphospholipid syndrome co-occurs in some cases, acute occlusion of the abdominal aorta often occurs due to embolization of intracardiac thrombi associated with atrial fibrillation [9, 10]. The patient in the present case had no history of atrial fibrillation, did not present with intracardiac thrombi, and exhibited co-occurring deep vein thrombosis. We therefore suspected the involvement of antiphospholipid syndrome. However, blood test results ruled out this possibility. Although the occurrence CCR-AAA followed by abdominal aortic occlusion suggests the involvement of both, there are no reports demonstrating a relationship between aortic rupture and aortic occlusion. Consequently, nothing is known about a causal relationship.

A ruptured abdominal aortic aneurysm is typically treated with open repair, although endovascular aneurysm repair has also recently been reported to be effective [11]. On the other hand, CCR-AAA has been treated in all reports with vascular prosthesis implantation or bypass surgery [1, 2, 4–6]. As in the present case, endovascular aneurysm repair is difficult to perform in cases with arterial occlusion. Open surgery for diagnosis and treatment is considered suitable for cases in which the possibility of a tumor or infection cannot be ruled out before surgery.

## Conclusion

For patients who present with a mass peripheral to the abdominal aorta, it may be necessary to conduct detailed examinations to discern not only the possibility of a tumor but also of infection and CCR-AAA.

## Data Availability

None.

## Abbreviations

AAA: Abdominal aortic aneurysm
CCR: Chronic contained rupture
CT: Computed tomography
FDG: Fluorine-18-fluorodeoxyglucose
MRI: Magnetic resonance imaging
PET: Positron emission tomography
